# Acid-base fractions separated from *Streblus asper* leaf ethanolic extract exhibited antibacterial, antioxidant, anti-acetylcholinesterase, and neuroprotective activities

**DOI:** 10.1186/s12906-018-2288-4

**Published:** 2018-07-24

**Authors:** Anchalee Prasansuklab, Atsadang Theerasri, Matthew Payne, Alison T. Ung, Tewin Tencomnao

**Affiliations:** 1 Program in Clinical Biochemistry and Molecular Medicine, Department of Clinical Chemistry, Faculty of Allied Health Sciences, Chulalongkorn University, Bangkok, 10330 Thailand; 2 School of Mathematical and Physical Sciences, Faculty of Science, The University of Technology Sydney, Sydney, NSW 2007 Australia; 3 Age-Related Inflammation and Degeneration Research Unit, Department of Clinical Chemistry, Faculty of Allied Health Sciences, Chulalongkorn University, Bangkok, 10330 Thailand

**Keywords:** Acid-base extraction, *Streblus asper*, Glutamate toxicity, HT22 cells, Neuroprotection, Alzheimer’s disease, Neurodegenerative diseases

## Abstract

**Background:**

*Streblus asper* is a well-known plant native to Southeast Asia. Different parts of the plant have been traditionally used for various medicinal purposes. However, there is very little scientific evidence reporting its therapeutic benefits for potential treatment of Alzheimer’s disease (AD). The study aimed to evaluate antibacterial, antioxidant, acetylcholinesterase (AChE) inhibition, and neuroprotective properties of *S. asper* leaf extracts with the primary objective of enhancing therapeutic applications and facilitating activity-guided isolation of the active chemical constituents.

**Methods:**

The leaves of *S. asper* were extracted in ethanol and subsequently fractionated into neutral, acid and base fractions. The phytochemical constituents of each fraction were analyzed using GC-MS. The antibacterial activity was evaluated using a broth microdilution method. The antioxidant activity was determined using DPPH and ABTS radical scavenging assays. The neuroprotective activity against glutamate-induced toxicity was tested on hippocampal neuronal HT22 cell line by evaluating the cell viability using MTT assay. The AChE inhibitory activity was screened by thin-layer chromatography (TLC) bioautographic method.

**Results:**

The partition of the *S. asper* ethanolic leaf extract yielded the highest mass of phytochemical constitutions in the neutral fraction and the lowest in the basic fraction. Amongst the three fractions, the acidic fraction showed the strongest antibacterial activity against gram-positive bacteria. The antioxidant activities of three fractions were found in the order of acidic > basic > neutral, whereas the decreasing order of neuroprotective activity was neutral > basic > acidic. TLC bioautography revealed one component in the neutral fraction exhibited anti-AChE activity. While in the acid fraction, two components showed inhibitory activity against AChE. GC-MS analysis of three fractions showed the presence of major phytochemical constituents including terpenoids, steroids, phenolics, fatty acids, and lipidic plant hormone.

**Conclusions:**

Our findings have demonstrated the therapeutic potential of three fractions extracted from *S. asper* leaves as a promising natural source for neuroprotective agents with additional actions of antibacterials and antioxidants, along with AChE inhibitors that will benefit in the development of new natural compounds in therapies against AD.

## Background

Herbal medicines are gaining significant attention globally in primary health care due to its various advantages over prescribed synthetic drugs, especially in long-term usage [1–3]. Synthetic FDA approved drugs, during long-term usage, may have adverse side effects [4, 5], and are not cost-effective or readily affordable in under developed and developing countries [6–10]. The use of medicinal plants or plant-derived substances in the prevention and treatment of various diseases including Alzheimer’s disease (AD) has been proven to be effective and is on the rise [11–15]. Two examples of plant-derived FDA approved drugs used for the treatment of AD are rivastigmine and galantamine, which were isolated from *Physostigma venenosum* and *Galanthus caucasicus,* respectively [16, 17]. Other promising natural products such as Huperzine A (derived from *Huperzia serrata*), curcumin (derived from *Curcuma longa*), and resveratrol (derived from *Vitis vinifera*) also possess excellent anti-AD activity and they are in Phase II or III clinical trials [18, 19]. Moreover, a number of medicinal plants with antioxidant, anti-inflammatory, and anti-apoptotic effects are currently being researched as an excellent source of neuroprotective agents and/or anti-AD drugs [16, 17, 20–23].

AD is one of the most common neurodegenerative diseases, characterized by the progressive loss of neuronal cells in the central nervous system which eventually contributes to memory impairment. Moreover, as the result of abnormal functioning in several areas of the patient’s brain in the late stage of disease, AD can be fatal and it is officially ranked as the sixth-leading cause of death in the United States (U.S.) in 2018 [24]. AD is suffered by 35–40 million patients worldwide and there is currently no proven complete cure [25]. Although the exact pathogenic mechanisms underlying AD remains unknown, the neurotransmitter acetylcholine and glutamate are potentially considered as therapeutic targets and have been focused since both neurotransmission systems were found aberrant in the brains of individuals with AD [26]. Drugs commonly used to treat AD include acetylcholinesterase (AChE) inhibitors (e.g., galantamine and donepezil) and *N*-methyl-D-aspartate (NMDA)-type glutamate receptor antagonists (e.g., memantine). Nevertheless, these drugs provide only symptomatic relief but not a cure. The debate over the therapeutic benefits versus the side effects and the financial cost of these drugs has continued for decades [27–29]. The discovery of therapeutic, cost effective AD drugs devoid of adverse side effects is a very active area of research [30].

Besides disturbances of neurotransmitters, oxidative stress and exacerbation of inflammatory responses have been associated with AD as significant contributors to neuronal damage and neurodegeneration [31–33]. Interestingly, bacterial infection is now considered as a risk or causative factor of AD by triggering chronic inflammation in the brain that may subsequently promote the initiation and progression of the disease [34–36]. Additionally, the presence of bacteria in the brain may play a role in the accumulation and deposition of amyloid beta (Aβ) peptides, the major pathological hallmark of AD, which these peptides may be recruited to the site of infection due to their functions in innate immunity as antimicrobials [37, 38]. A dramatic reduction of cerebral Aβ levels was observed when transgenic mouse model of AD was in the absence of gut microbiota [39] or in the prolonged shift of gut microbial composition induced by broad-spectrum combinatorial antibiotic treatment [40]. Recent evidence using next generation sequencing (NGS) analysis that observed an increase of bacterial populations in post-mortem brains from patients with AD, also supports the involvement of bacterial infection in AD [41]. Hence, controlling AD-associated infections by using compounds with bactericidal or antibacterial activities was highlighted as an alternative strategy for disease prevention and treatment [34, 35, 42, 43].

Medicinal plants can be of important natural resources for novel agents that may constitute an alternative to the present drugs used in the treatment of several illnesses including AD [11]. *Streblus asper* Lour. is a well-known medicinal plant that belongs to family Moraceae and distributes mainly over the region of Southeast Asia. Different parts of the plant have been traditionally used for various medicinal purposes such as treatment of fever, toothache, filariasis, leprosy, snakebite, diarrhoea, piles, epilepsy, epistaxis, heart disease, urinary tract complaints, stomachache, obesity, skin diseases, wounds, and cancer [44, 45]. It has also been used as an ingredient in a traditional Thai formula for longevity [46]. Studies on the crude plant extract as well as the isolated compounds have demonstrated that *S. asper* exhibits various pharmacological properties including antioxidant, anticancer [47], antimicrobial [48], antimalarial [49], anti-filarial [50], anti-inflammation [51], and anti-hepatitis B activities [52]. Moreover, the neuroprotective properties of *S. asper* leaf extracts in both in vivo and in vitro models of neurodegenerative diseases were recently reported [53, 54]. However, the active phytochemical ingredients responsible for neuroprotection have not been clearly identified. The main objective of our research is to enhance the utilization of this plant extract with the best efficacy for therapeutic applications and facilitate the clarification of its active components. This was achieved by first carrying out the fractionation of the crude ethanolic extract of *S. asper* leaves using liquid-liquid extraction based on pH properties of its phytochemical constituents. The resulting fractions of organic neutral, acidic, and basic components were investigated and compared to each other for their pharmacological potentials including antibacterial, antioxidant, anti-AChE, and neuroprotective activities.

## Methods

### Chemicals and reagents

Acetylcholinesterase from electric eel (EC 3.1.1.7, type V-S), bovine serum albumin (BSA), 1-naphthyl acetate, Fast Blue B salt, galantamine, Tris-HCl, dimethyl sulfoxide (DMSO), Dulbecco’s modified Eagle’s medium (DMEM), fetal bovine serum (FBS), 2,2′-Azino-bis(3-ethylbenzothiazoline-6-sulfonic acid) diammonium salt (ABTS), and 2,2-Diphenyl-1-picrylhydrazyl (DPPH) were purchased from Sigma-Aldrich (St. Louis, MO, USA). Phosphate buffer saline (PBS) and Penicillin/Streptomycin solution were purchased from Hyclone (Logan, Utah, USA). Trypsin-EDTA was purchased from Gibco (Waltham, MA, USA). 3-(4,5-dimetylthiazol-2-yl)-2,5-diphenyltetrazoliumbromide (MTT) was purchased from Bio Basic (Markham, Ontario, Canada). L-ascorbic acid was purchased from Calbiochem (San Diego, CA, USA). Mueller-Hinton broth was purchased from Himedia laboratories (Mumbai, MH, India). Other reagents used in extraction process were of analytical grade.

### Plant collection and identification

Leaves of *S. asper* were collected from the Princess Maha Chakri Sirindhorn Herbal Garden (Rayong Province, Thailand). The plant was authenticated by Professor Dr. Thaweesakdi Boonkerd and deposited in the herbarium of Kasin Suvatabhandhu (Department of Botany, Faculty of Science, Chulalongkorn University, Thailand) under voucher number A013419 (BCU).

### Preparation of crude extract

After collection, the ethanolic extract of *S. asper* leaves was prepared as previously described [54]. In brief, the dry powered plant material (0.73 kg) was soaked in 100% ethanol (7.3 L) with a 1:10 sample-solvent ratio for 48 h at room temperature (RT) in the dark with continuous shaking. The resulting liquid extract was filtered and concentrated using rotary evaporator to give 29.44 g of crude extract. Part of this concentrated extract was dissolved in DMSO, passed through a 0.2-μm filter, and stored at − 20 °C as a stock solution for evaluating biological activities.

### Preparation of acid-base fractions

**Fig. 1:**
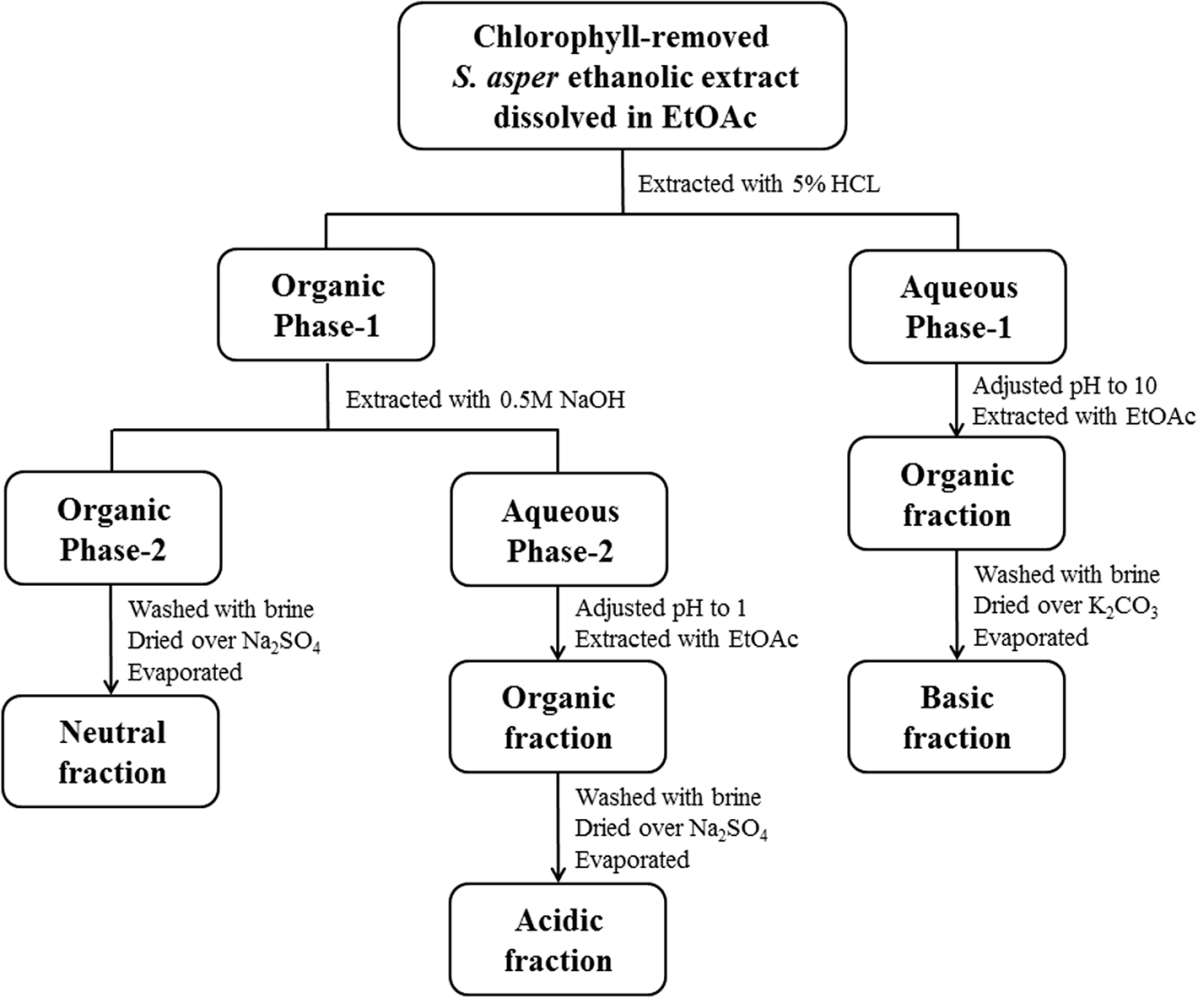
Acid-Base extraction of *S. asper* ethanolic extract

For efficient acid-base extraction, crude ethanolic extract of *S. asper* leaves (29.44 g) was firstly dissolved in ethyl acetate (EtOAc) (700 mL) and partitioned with distilled water (700 mL). The resulting organic solution was concentrated, re-dissolved in dioxane (200 mL) and slowly added dropwise of distilled water (80 mL) while stirring continuously to induce the precipitation of chlorophyll [55]. Then, the precipitates were removed by three rounds of centrifugation (4400 rpm for 20 min) with each round being followed by filtration through filter papers. The chlorophyll-removed extracts were combined, and solvents were removed to give a thick paste. The paste was used for fractionation into neutral, acidic, and basic fractions based on the method of alkaloid extraction previously used by Mungkornasawakul et al. [56], with some modifications. The procedure of acid-base extraction was summarized in Fig. 1. The crude paste was dissolved in EtOAc (150 mL) and extracted with 5% hydrochloric acid (HCl) (700 mL). The ethyl acetate (Organic phase-1) layer was separated from the 5% HCl (Aqueous phase-1) layer and set aside for basic extraction. The 5% HCl solution was made basic (pH 10) with 10 M sodium hydroxide (NaOH) and the resulting solution was further extracted with EtOAc (200 mL × 2). The ethyl acetate extracts were washed with saturated NaCl solution and dried over anhydrous potassium carbonate (K_2_CO_3_). The solvent was removed to afford the basic fraction.

Simultaneously, ethyl acetate (Organic phase-1) layer was extracted with an equal volume of 0.5 M NaOH solution. The top organic layer (Organic phase-2) was separated from the aqueous NaOH layer (Aqueous phase-2), then washed with a saturated solution NaCl and dried over anhydrous sodium sulfate (Na_2_SO_4_) and the solvent was removed under reduced pressure to afford the neutral fraction. Finally, the remaining the aqueous NaOH layer (Aqueous phase-2) was then acidified to pH 1 with 5% HCl and extracted with EtOAc (200 mL × 2). The combined ethyl acetate extracts were washed with a saturated solution NaCl and dried over anhydrous Na_2_SO_4_, and the solvent was removed under reduced pressure to afford the acidic fraction. Parts of three fractions obtained after acid-base extraction were dissolved in DMSO, filtered through a 0.2-μm filter, and stored at − 20 °C as a stock solution for evaluating biological activities.

### GC-MS analysis

Analysis of volatile chemical constituents was performed using Agilent 6890 gas chromatograph fitted with an Agilent HP-5 MS fused-silica capillary column (5% polysilphenylene, 95% polydimethylsiloxane column, 30 m × 0.25 mm, i.d. 0.25 μm film thickness), coupled to an Agilent 5973 N mass selective detector with electron-ionization (EI) source (Agilent Technologies, Palo Alto, CA, USA). The samples were dissolved in ethyl acetate and injected automatically in the split mode with a split ratio of 1:25 and injection volume of 1 μL. Helium was used as the carrier gas at a constant flow rate of 1.2 mL/min. The total run time was 10.8 min. The column oven temperature was programmed initially at 50 °C for 2 min. Then, it was raised to 290 °C with a rate of 50 °C/min and held at 290 °C for 4 min. The mass spectrometer was operated with ionization energy of 70 eV, ion source temperature of 230 °C, detector temperature of 150 °C, and in the scan mode over the mass range of m/z 40–450. The compounds were identified by matching their recorded retention times and mass spectral patterns with the NIST mass spectral library (NIST08.L).

### Determination of antibacterial activity

The antibacterial activity was determined by broth microdilution according to CLSI guidelines. The test medium was cation-adjusted Mueller-Hinton broth (CAMHB). Test Medium was used for *S. aureus* ATCC25923, *B. subtilis* SU5, *E. coli* MG1655 and *P. aeruginosa* PA14. Serially diluted concentrations of samples or standard antibiotic ampicillin (a positive inhibition control) were tested using a 96 well plate, which was inoculated with a bacterium cell concentration of approximately 5 × 10^5^ CFU. The CAMHB medium plus sample without inoculum was used as a negative control. After 20 h of incubation at 37 °C statically, the minimum inhibitory concentration (MIC) was defined as the lowest concentration of compound that showed 95% cell growth inhibition, determined by measuring absorbance at 595 nm. The experiment was performed in biological triplicates.

### Determination of antioxidant activity

The antioxidant activity was evaluated in vitro using the DPPH and ABTS radical scavenging assays modified for a 96-well microtiter plate format as described previously [54]. Briefly, various concentrations of samples or ascorbic acid (a positive control) in absolute ethanol and the DPPH• or ABTS• + working solution were mixed in a ratio of 9:1 (*v*/v) in a 96 well-plate. The working solution plus absolute ethanol was used as a negative control. The reaction mixture was incubated in the dark at RT for 15 min or 30 min and the absorbance was recorded using a microplate reader (BioTek Instruments, Winooski, VT, USA) at 517 nm or 734 nm for DPPH or ABTS assay, respectively. Radical scavenging activity was calculated as the percent inhibition of free radicals using the following equation: % Inhibition = 100 - [(Abs of the sample - Abs of blank) × 100/ Abs of control]. The antioxidant capacity of each sample was also compared with those of ascorbic acid (vitamin C) and was expressed as vitamin C equivalent antioxidant capacity (VCEAC) in mg per g of dry weight sample.

### Determination of anti-acetylcholinesterase activity

The AChE inhibitory activity was screened by using thin-layer chromatography (TLC)-direct bioautographic assay adapted from Marston’s method [57]. This assay is based on the activity of AChE in converting the substrate 1-naphthyl acetate to 1-naphthol, which in turn reacts with the chromogenic agent Fast Blue B salt to produce a purple-coloured diazonium dye. The working solution of AChE was prepared by dissolving 1.21 mg of lyophilized powder of AChE from electric eel in 135 mL of 0.05 M Tris-HCl buffer at pH 7.8 with 150 mg of bovine serum albumin. The silica gel F_254_ TLC plates (Merck, Darmstadt, Germany) were prewashed with acetone and thoroughly dried before use. Briefly, 1 mg of each sample was dissolved in ethyl acetate (1000 ppm), 6.7 μL (6.7 μg) of the solution was spotted on a pre-washed TLC plate and developed with the solvent system of hexane: ethyl acetate (7:3, *v*/v). Then, 3.3 μL (0.3 μg) of galantamine solution (0.1 mg/mL, 100 ppm) was also applied on the plate as a positive control. After completely air-dried, the TLC plates were then saturated by spraying with the enzyme stock solution (6.71 U/mL) and incubated in a humidified chamber for 20 min at 37 °C. Afterwards, the enzyme activity was detected by spraying with the mixture solution containing a part of 13.8 mM of 1-naphthyl acetate in ethanol plus four parts of 5.27 mM of Fast Blue B salt in distilled water, onto the moist TLC plates. The presence of potential compounds with anti-AChE activity was determined as the appearance of white (clear) spot on the purple-coloured background.

### Cell culture and treatments

Immortalized mouse hippocampal HT22 cell line, a kind gift from Prof. David Schubert at the Salk Institute (San Diego, CA, USA), was grown in DMEM supplemented with 10% FBS and 1% Penicillin/Streptomycin solution. The cells were maintained at 37 °C in a 5% CO_2_ humidified incubator and sub-cultured when they reach about 80–90% confluency. For cell viability assay, HT22 cells were seeded onto a 96-well microtiter plate at a density of 6 × 10^3^ cells per well and allowed to attach. Twenty-four hours later, the cells were incubated for additional 18 h with the medium in the absence or presence of 5 mM glutamate or in the presence of 5 mM glutamate plus various concentrations of tested extracts.

### Determination of cell viability

The cell viability of HT22 cells was measured by the MTT colourimetric assay to determine the neuroprotective property of tested extracts against glutamate cytotoxicity. In the assay, untreated HT22 cells served as a negative control, while cells treated with 100% DMSO served as a positive control. MTT was prepared as a stock solution of 5 mg/ml in PBS, pH 7.2, sterilized through a 0.2-μm filter, and stored at − 20 °C. At the end of incubation time, 20 μL of MTT solution was added to each well, followed by incubation at 37 °C for 4 h. The medium containing MTT solution was then removed and 100 μL of DMSO was replaced in each well to dissolve the purple formazan crystals produced from viable cells. The absorbance was read by a microplate reader (BioTek Instruments, Winooski, VT, USA) at 550 nm. The percentage of cell viability was calculated by the following formula: Cell viability (%) = [Abs of treated cells/ Abs of untreated cells (control)] × 100.

### Statistical evaluation

For antioxidant and cell viability assays, the experiments were performed at least in triplicate and the data are represented as means ± standard deviation (SD) or means ± standard error of the mean (SEM) as indicated in figures. The statistical analyses were performed using SPSS software version 17.0 (SPSS Inc., Chicago, IL, USA). Pearson’s correlation test was conducted to evaluate the relationship between two antioxidant assays. One-way analysis of variance (ANOVA), followed by the post hoc Tukey HSD multiple comparison tests was employed to determine the differences among group means. *P* values < 0.05 were considered statistically significant.

## Results

### Extraction yields and the chemical composition of the *S. asper* fractions obtained by acid-base extraction

**Fig. 2:**
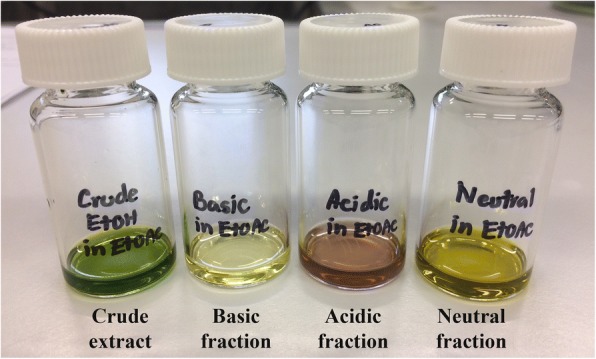
Crude ethanolic extract of *S. asper* leaves and its acid-base fractions

Acid-base extraction of 29.44 g of the *S. asper* ethanolic leaf extract yielded 0.158 g (0.54%, *w*/w) of basic fraction, 0.476 g (1.62%, w/w) of acidic fraction, and 1.128 g (3.83%, w/w) of neutral fraction. Figure 2 shows the color of crude leaf extract and the fractions obtained. The brownish and yellowish colors observed in the acid-base fractions suggested that most of the chlorophyll (green pigment) in the leaves was successfully removed from crude leaf extract. Each fraction was further analyzed by GC-MS and tentative compounds were identified by comparing their GC retention indices and mass spectral patterns with the database at the match quality value above 80%. A total of 11 tentative volatile compounds were proposed including terpenoids, steroids, phenolics, fatty acids, nitrogen-sulfur containing compounds, as well as lipidic plant hormone were listed in Table 1.

**Table 1:** GC-MS analysis of the volatile components presented in the *S. asper* fractions

Fraction	Retention time (min)	Relative area (%)	Tentative identification	Match quality (%)
Neutral	6.111	2.6	Dihydroactinidiolide	94
	6.548	5.5	Cadalene	80
	6.696	11.7	n.i.	–
	7.124	7.0	Benzothiazole, 2-(2-hydroxyethylthio)-	99
	7.466	5.9	n.i.	–
	8.370	9.4	Cholest-14-en-3-ol, 4-methyl-, (3.beta.,4.alpha.,5.alpha.)-	93
	8.417	10.8	n.i.	–
	8.612	7.4	2,4-Bis(1-phenylethyl)phenol	86
	10.072	7.3	n.i.	–
Acidic	5.531	0.6	4-Hydroxybenzaldehyde	91
	5.683	0.2	Vanillin	96
	6.477	0.3	(+/−)-Jasmonic acid	95
	6.543	3.6	Cadalene	83
	6.686	3.1	n.i.	–
	7.010	2.2	Palmitic acid	99
	7.114	6.9	Benzothiazole, 2-(2-hydroxyethylthio)-	98
	7.457	12.6	Linolenic acid	99
	7.485	8.5	n.i.	–
	8.355	6.8	Cholest-14-en-3-ol, 4-methyl-, (3.beta.,4.alpha.,5.alpha.)-	91
	8.398	7.2	n.i.	–
	8.593	5.8	2,4-Bis(1-phenylethyl)phenol	93
Basic	5.421	1.6	n.i.	–
	6.543	2.2	Phenol, 2-(1-phenylethyl)-	91
	6.686	30.1	n.i.	–
	6.762	4.9	n.i.	–
	7.109	6.0	Benzothiazole, 2-(2-hydroxyethylthio)-	99
	7.295	8.2	n.i.	–
	7.471	12.4	n.i.	–
	8.350	4.2	n.i.	–
	8.398	4.6	n.i.	–
	8.588	3.3	Phenol, 2,4-bis(1-phenylethyl)-	91

*n.i.* Not identified

### Antibacterial activity of the *S. asper* fractions

The antibacterial activity of the crude *S. asper* ethanolic extract and its acid-base fractions are presented in Table 2. Amongst the three fractions examined, the acidic fraction exhibited the strongest antibacterial activity against two gram-positive bacteria *S. aureus* and *B. subtilis*, with a MIC value of 125 μg/mL. However, at the highest tested concentration of 1000 μg/mL, none of the three fractions showed inhibition of bacterial growth at ≥95% against the two gram-negative bacteria *E. coli* and *P. aeruginosa*.

**Table 2:** Antibacterial activities of crude ethanolic extract of *S. asper* leaves and its fractions against a range of microorganisms determined by the broth microdilution method

Microorganisms	Minimum inhibitory concentration (MIC) in μg/mL			
	Crude extract	Neutral fraction	Acidic fraction	Basic fraction
Gram-positive bacteria				
*Staphylococcus aureus* (ATCC25923)	1000	1000	125	1000
*Bacillus subtilis* (SU5)	1000	250	125	500
Gram-negative bacteria				
*Escherichia coli* (MG1655)	> 1000	> 1000	> 1000	> 1000
*Pseudomonas aeruginosa* (PA14)	> 1000	> 1000	> 1000	> 1000

MIC values are the lowest concentrations at which at least 95% bacterial growth reduction

The tested concentration of samples ranged from 125 to 1000 μg/mL

### Antioxidant capacity of the *S. asper* fractions

**Fig. 3:**
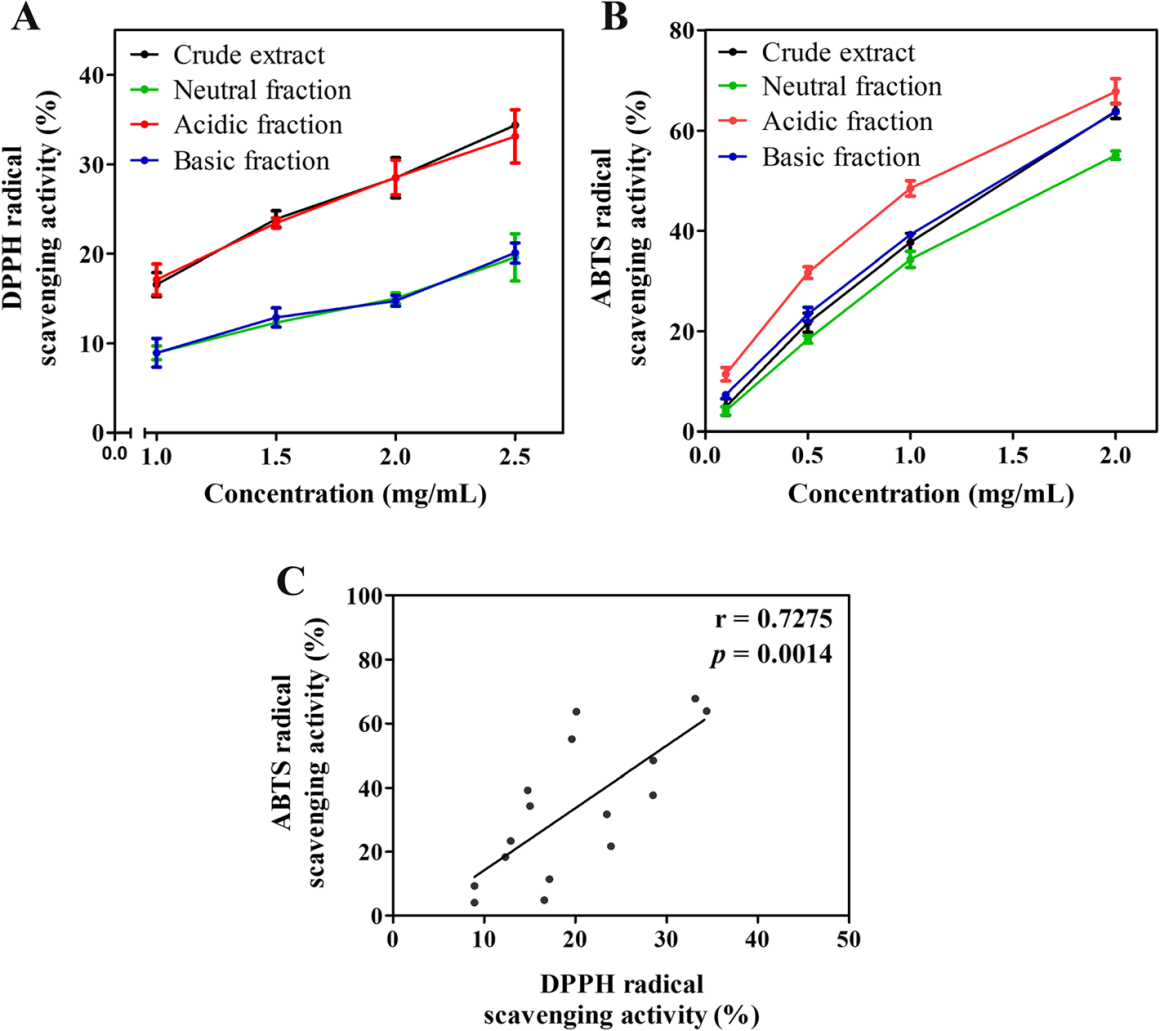
The dose-response scavenging effects of the crude ethanolic extract of *S. asper* leaves and its fractions on **a** DPPH and **b** ABTS free radicals. Data are expressed as means ± SD of 3–7 replicates. **c** Pearson’s correlation analysis between DPPH and ABTS scavenging activities based on mean values of all samples analyzed

The DPPH radical scavenging activities at 1 mg/mL concentration of the crude *S. asper* ethanolic extract and its acid-base fractions varied ranging from 8.94 to 17.16% (10.00 to 18.88 mg VCEAC/g dry weight), whereas their scavenging activities on ABTS radical were found much higher than those on DPPH radical in the range of 34.29 to 48.45% (29.02 to 39.45 mg VCEAC/g dry weight) (Table 3). All the crude extract and its acid-base fractions exhibited concentration-dependent scavenging effects towards the DPPH (Fig. 3a) and ABTS radicals (Fig. 3b). Amongst all the extract and three fractions examined, the acidic fraction possessed the strongest antioxidant capacity, while the neutral fraction showed relatively weak antioxidant property based on the results from both DPPH and ABTS assays. The descending order of antioxidant potential among three isolated fractions was as acidic > basic > neutral. Pearson’s correlation revealed a significant positive moderate relationship (*r* = 0.7275, *p* = 0.0014) between the DPPH and ABTS antioxidant activities of all tested samples (Fig. 3c).

**Table 3:** Antioxidant capacities of the crude ethanolic extract of *S. asper* leaves and its fractions determined by the DPPH and ABTS scavenging assays

Sample	DPPH scavenging assay	ABTS scavenging assay		
	%Radical Scavenging activity (of 1 mg/mL sample)	mg VCEAC/g dry weight sample	%Radical Scavenging activity (of 1 mg/mL sample)	mg VCEAC/g dry weight sample
Crude extract	16.58 ± 1.33^a^	18.37 ± 1.25^a^	37.69 ± 1.81^a^	31.52 ± 1.63^a^
Neutral fraction	8.94 ± 0.78^b^	10.63 ± 1.38^b^	34.29 ± 1.56^b^	29.02 ± 1.43^b^
Acidic fraction	17.16 ± 1.74^a^	18.88 ± 1.39^a^	48.45 ± 1.55^c^	39.45 ± 1.43^c^
Basic fraction	8.94 ± 1.62^b^	10.00 ± 0.77^b^	39.23 ± 0.66^a^	32.66 ± 0.84^a^

Results are expressed as mean ± SD of at least three replicates

Different superscript letters in the same column indicate a significant difference between the means by one-way ANOVA (*p* < 0.05) and the same letter indicates that there is no statistical difference

### Neuroprotective activity of the *S. asper* fractions

**Fig. 4:**
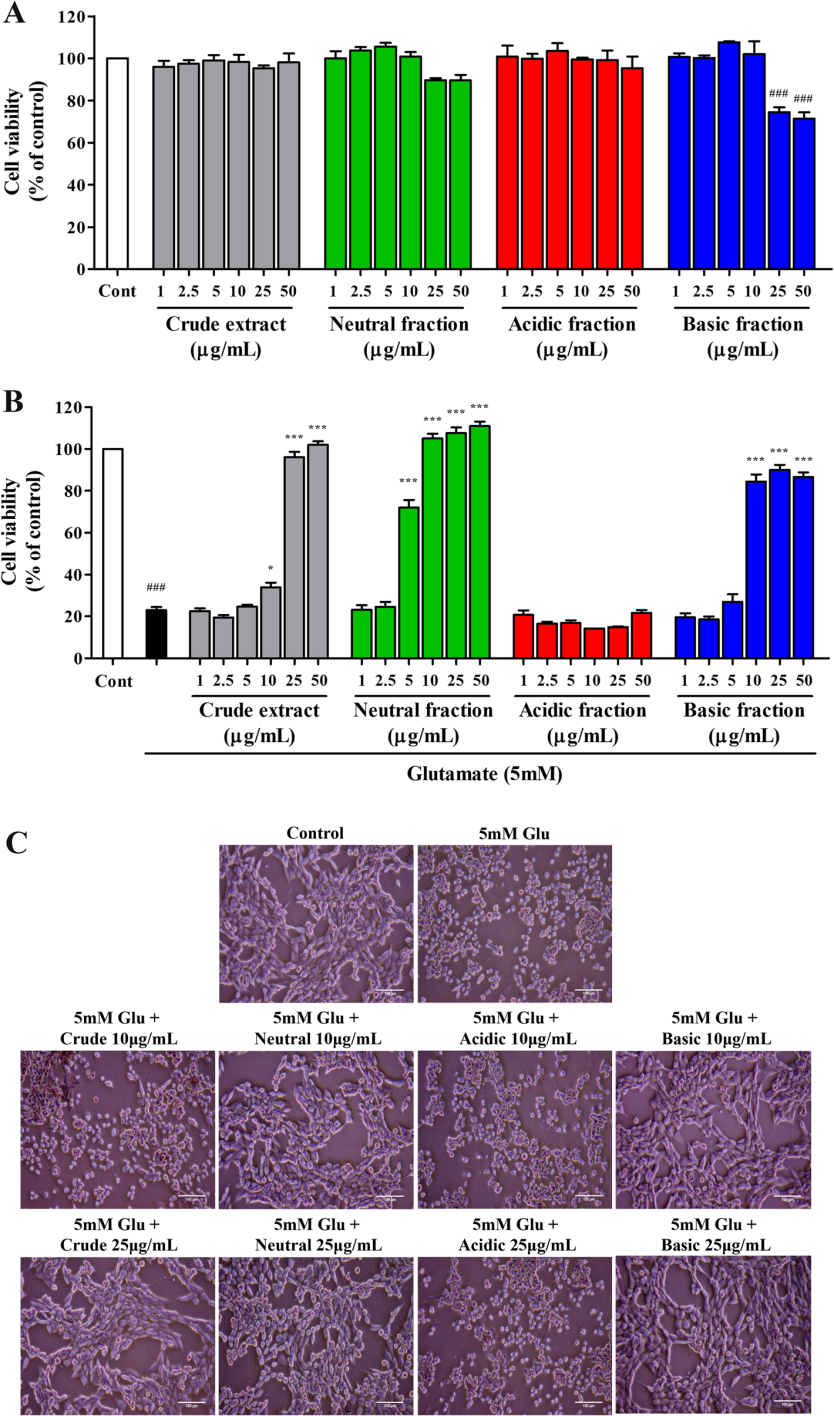
Protective effects of the crude ethanolic extract of *S. asper* leaves and its fractions against glutamate-induced neuronal cell death. **a** Relative MTT viability of HT22 cells exposed to various concentrations of extracts. ^*###*^*P* = 8.8 × 10^− 5^ for 25 μg/mL and ^*###*^*P* = 7.5 × 10^− 6^ for 50 μg/mL of basic fraction vs. control. **b** Relative MTT viability of HT22 cells exposed to glutamate alone or glutamate combined with different concentrations of extracts. ^*###*^*P* = 2.9 × 10^− 11^ vs. control; *P = 3.1 × 10^− 2^ and ***P = 2.9 × 10^− 11^ vs. glutamate-treated cells. **c** Representative morphological images at 5X magnification (scale bar = 100 μm) of untreated HT22 cells (control), or cells treated with glutamate alone, or with glutamate plus crude extract or fractions at 1 and 10 μg/mL. Data are expressed as means ± SEM of 4 independent experiments with 2–3 replicates each

The neuroprotective properties of the crude *S. asper* ethanolic extract and its acid-base fractions were evaluated against glutamate toxicity in hippocampal neuronal HT22 cells. The crude extract, neutral and acidic fractions did not exhibit a noticeable cytotoxic effect on HT22 cells whose cell viabilities were above 90% following exposure to varying concentrations ranging from 1 to 50 μg/mL (Fig. 4a). The basic fraction became toxic to the cells when the concentration was increased above 25 μg/mL. Treatment of HT22 cells with 5 mM glutamate significantly reduced cell viability by approximately 80%. However, co-treatment with crude extract, either neutral fraction or basic fraction significantly increased the viability of glutamate-treated HT22 cells in a dose-dependent manner, as determined using MTT assay (Fig. 4b) and examined morphologically under a phase contrast microscope (Fig. 4c). The cytotoxic effect of glutamate could be considerably restored (comparable to about 80% of the control level) in the presence of the crude extract, neutral fraction, and the basic fraction at the minimum concentration of 25, 5, and 10 μg/mL, respectively. The acidic fraction did not show any protective effects against glutamate-induced cytotoxicity.

### Anti-acetylcholinesterase activity of the *S. asper* fractions

**Fig. 5:**
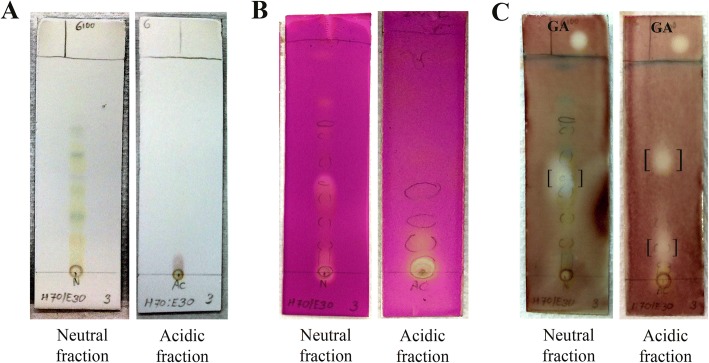
Anti-AChE activities of neutral and acidic fractions obtained from *S. asper* leaves. TLC chromatograms of neutral and acidic fractions (at 6.7 μg application) in a solvent system of hexane:ethyl acetate (7:3, *v*/v) were observed **a** under visible light, **b** by staining with KMnO_4_, and **c** by bioautographic method for screening on AChE inhibitory activity, where white spots against the dark background represent the inhibition. Galantamine (GA) was used as a positive control at 0.3 μg. Brackets indicate AChE inhibiting constituents

TLC chromatogram of the neutral fraction isolated at least 9 visible spots of constituents under visible light (Fig. 5a) and confirmed by visualizing under short-wavelength UV radiation (254 nm), in which three of them were also observed by KMnO_4_ staining (Fig. 5b). However, in the acidic fraction, there was only one spot visible under visible light (Fig. 5a), while three more spots could be only viewed under UV light, in which one of them was also observed by KMnO_4_ staining (Fig. 5b). Using a similar solvent system to that employed in the experiment mentioned above, the AChE inhibitory properties of the neutral and acidic fractions were further evaluated on TLC plates by the bioautographic method and are shown in Fig. 5c. In this assay, the white spot of inhibition on a dark purple background of the plate represented the chemical components with AChE inhibitory activity. A total of three chemical constituents corresponding to AChE inhibitory activity in the neutral and acidic fractions were found. TLC bioautogram of the neutral fraction showed one spot of inhibition with R_f_ value of 0.44 and positive staining for KMnO_4_ (Fig. 5b and c). TLC bioautogram of the acidic fraction showed two spots of inhibition with R_f_ values of 0.15 and 0.55. The spot with R_f_ 0.15 was also positively stained with KMnO_4_ (Fig. 5b and c).

## Discussion

The current available medications can only reduce the severity of AD in certain patient groups, since there has been no complete cure known for it. Importantly, AD can be fatal and is now recognized as one of the major global health problems [24]. Therefore, its prevalence continues to rise rapidly, along with the burden on public healthcare costs. This has a long-term impact on the socio-economic development of the developing countries. In challenging this difficult situation, a number of researchers have put a considerable effort for decades in search of alternative treatment methods. Research in the field of herbal medicines has attracted much attention in recent years, as it is believed to have advantages over modern synthetic medicines with regard to efficacy, safety, and cost for treatment of various medical problems. In Southeast-Asian countries, *S. asper* is a well-known medicinal plant that has been traditionally used for a variety of illness conditions despite limited scientific evidence [44–46]. Interestingly, the ethanolic leaf extracts of this plant have shown pharmacological properties including antibacterial, neuroprotective, and cognitive-improving effects that can be beneficial for the treatment of AD [48, 53, 54]. However, the active constituents responsible for those different activities have not been clearly identified. In this study, the *S. asper* leaf ethanolic extract was fractionated into fractions based on pH properties and further evaluated for their antibacterial, antioxidant, anti-AChE, and neuroprotective activities. These biological activities could enhance the therapeutic applications and facilitating activity-guided isolation of active compounds of the *S. asper* extract in the near future.

*S. asper* is commonly used to treat skin infections (e.g. boils, leprosy, and wounds) and has beneficial role for oral health and hygiene (e.g. relief of toothache, antigingivatis, and strengthening teeth and gum) [44]. The bacterial species, which are most frequently found associated with AD, are commonly found in oral cavities such as spirochetes [42] and actinobacteria [41], thus it is tempting us to investigate the antibacterial properties of this plant. In this study, the crude ethanolic extract was found to inhibit cell growth of gram-positive bacteria, *S. aureus* and *B. subtilis*. This effect was more profound when the extract was fractionated to the acidic fraction (MIC of 125 μg/mL) with at least eight-fold higher than to ethanol crude extract. Neither the ethanol extract nor acid-base fractions inhibit the growth of gram-negative bacteria, *E. coli* and *P. aeruginosa*. Previous work by Wongkam et al. on the crude 50% ethanolic extract, reported no antibacterial activity when tested on the bacteria strains used in our study. Their study instead showed weak MIC and MBC of 1.93 mg/mL against *Streptococcus mutans, Porphyromonas gingivalis, and Actinobacillus actinomycetemcomitans;* bacteria that are commonly associated with dental caries and gingivitis [48, 58, 59]. The source of plant collection and the percentage of ethanol used for extraction may be the critical factors for the different findings. Although gram-negative bacteria are generally more harmful, some of the gram-positive bacteria can also be pathogenic in humans. *S. aureus* is one of the most common causes of skin and nosocomial infections as well as other infections at various sites of the body (e.g. bone, joint, lung, and gastrointestinal tract) with symptoms ranging from minor to life-threatening [60]. Here, our study provides supporting evidence for the use of acidic fraction from SA leaves to treat gram-positive bacterial infections particularly caused by *S. aureus*. Moreover, the promising antibacterial activity of the acidic fraction relative to other fractions could be contributed by the action of its major volatile component, linolenic acid (Table 1, [54]). It has been demonstrated that long-chain unsaturated fatty acids including linolenic acid exerted highly potent activity against gram-positive bacteria [61–64].

Free radicals are groups of atoms with an unpaired number of electrons residing in the outermost shell. The majority of free radicals are generated from oxygen molecules and called reactive oxygen species (ROS). Once formed in excess, these highly reactive free radicals can cause damage, in the process called oxidative stress, to important cellular components such as lipids, proteins and nucleic acids, subsequently, alter the structures and functions that are associated with various human diseases including AD [65]. In general, these detrimental effects of free radicals can be counterbalanced by antioxidants, which are naturally derived from plants. *S. asper* can be considered as an alternative source for antioxidants since this plant has been shown promising antioxidant properties in both in vitro and in vivo [54, 66–70]. In line with the previous reports, our present study also revealed the antioxidant properties, like free radical scavenging, in *S. asper* leaf extracts. The ethanol crude extract, neutral, acidic and basic fractions exhibited relatively two- to four-fold higher activities in the ABTS assay than in the DPPH assay and the percentage of scavenging activities determined in both assays were moderately correlated (*r* = 0.73). The slight difference in results could be probably due to the distinct solubility of tested substances in water, as hydrophilic antioxidants were found better reflected by the ABTS than the DPPH assay [71, 72]. Interestingly, after fractionation of crude ethanolic leaf extract, the scavenging activities on DPPH and ABTS radicals were found significantly higher in the acidic fraction when compared to the crude extract and other fractions, while the neutral fraction showed the lowest antioxidant capacity in both assays. These findings suggest that the components with antiradical activity in *S. asper* leaves are majorly in acidic and water-soluble forms. Nevertheless, the DPPH and ABTS radicals used in this study are uncommonly found in the human body, the antioxidant capacity has to be further investigated using ROS, which are produced as by-products during cellular metabolism such as superoxide radicals (O_2_•−), hydrogen peroxide (H_2_O_2_), or highly reactive hydroxyl radicals (OH•) for a better idea.

In an effort to develop a more effective treatment for AD, several studies have been searching for new therapeutic targets underlying the disease pathology. Apart from the cholinergic hypothesis that has been proposed in the etiology of AD for several decades [73], growing evidence links oxidative glutamate toxicity (or oxytosis) to AD by supporting that glutamate-induced neuronal cell death via non-receptor-mediated oxidative toxicity pathway involves in the pathogenic mechanism of neurodegeneration [74–76]. Hence, targeting towards the glutamate-mediated oxidative toxicity pathway may offer a new approach for treating AD as well as other neurodegenerative diseases. In accordance with our previous results, the present study demonstrated that crude ethanolic extract from *S. asper* leaves exerted neuroprotective property against glutamate-induced HT22 neuronal cell death [54]. This protective activity was also found higher following fractionation of crude ethanolic leaf extract. The strongest protective activity against glutamate toxicity in HT22 cells was observed in the neutral fraction, wherein the minimum concentration showing recovery of 80% cell viability was five-fold lower than that of the crude extract. Interestingly, TLC bioautography also suggested that neutral fraction contains at least one compound with AChE inhibitory activity with minimum inhibitory concentration requirement of approximately 6.7 μg. Therefore, this finding indicates that neutral fraction can be further developed as a potential multi-target agent for AD treatment. However, due to the weak antiradical activity of neutral fraction, it may exert neuroprotective capacity against glutamate-induced oxidative damage via other antioxidative mechanisms. For instance, induction of nuclear translocation of nuclear factor erythroid 2-related factor 2 (Nrf2) is a protective pathway proposed in our previous work [54].

## Conclusions

Our findings have demonstrated the therapeutic potential of three acid-base fractions extracted from the leaf part of *S. asper* as promising natural source for neuroprotective agents with additional actions of antibacterials and antioxidants, along with AChE inhibitors. Further studies should aim to isolate bioactive substances from neutral and acidic fractions and identify the mechanisms underlying their actions, which can be used to develop new natural agents as therapeutic drugs for the treatment of AD.

## Abbreviations

ABTS: 2,2′-Azino-bis(3-ethylbenzothiazoline-6-sulfonic acid) diammonium salt
AChE: Acetylcholinesterase
AD: Alzheimer’s disease
DPPH: 2,2-Diphenyl-1-picrylhydrazyl
GC-MS: Gas chromatography-mass spectrometry
MIC: Minimum inhibitory concentration
MTT: 3-(4,5-dimetylthiazol-2-yl)-2,5-diphenyltetrazoliumbromide
NMDA: *N*-methyl-D-aspartate
Nrf2: Nuclear factor erythroid 2-related factor 2
TLC: Thin-layer chromatography
VCEAC: Vitamin C equivalent antioxidant capacity
